# Interplay between Mast Cells and Regulatory T Cells in Immune-Mediated Cholangiopathies

**DOI:** 10.3390/ijms23115872

**Published:** 2022-05-24

**Authors:** Natalia M. Krajewska, Rémi Fiancette, Ye H. Oo

**Affiliations:** 1 Centre for Liver and Gastrointestinal Research & NIHR Birmingham Liver Biomedical Research Unit, Institute of Biomedical Research, Institute of Immunology and Immunotherapy, University of Birmingham, Birmingham B15 2TT, UK; r.b.fiancette@bham.ac.uk; 2 Centre for Rare Diseases, European Reference Network Rare Liver Centre, University Hospital Birmingham NHS Foundation Trust, Birmingham B15 2TH, UK; 3 Advanced Cellular Therapy Facility, University of Birmingham, Birmingham B15 2TT, UK; 4 Liver Transplant and Hepatobiliary Unit, Queen Elizabeth Hospital, University Hospital Birmingham NHS Foundation Trust, Birmingham B15 2TH, UK

**Keywords:** primary biliary cholangitis, primary sclerosing cholangitis, immune tolerance, inflammation, plasticity

## Abstract

Immune-mediated cholangiopathies are characterised by the destruction of small and large bile ducts causing bile acid stasis, which leads to subsequent inflammation, fibrosis, and eventual cirrhosis of the liver tissue. A breakdown of peripheral hepatic immune tolerance is a key feature of these diseases. Regulatory T cells (Tregs) are a major anti-inflammatory immune cell subset, and their quantities and functional capacity are impaired in autoimmune liver diseases. Tregs can undergo phenotypic reprogramming towards pro-inflammatory Th1 and Th17 profiles. The inflamed hepatic microenvironment influences and can impede normal Treg suppressive functions. Mast cell (MC) infiltration increases during liver inflammation, and active MCs have been shown to be an important source of pro-inflammatory mediators, thus driving pathogenesis. By influencing the microenvironment, MCs can indirectly manipulate Treg functions and inhibit their suppressive and proliferative activity. In addition, direct cell-to-cell interactions have been identified between MCs and Tregs. It is critical to consider the effects of MCs on the inflammatory milieu of the liver and their influence on Treg functions. This review will focus on the roles and crosstalk of Tregs and MCs during autoimmune cholangiopathy pathogenesis progression.

## 1. Introduction

Cholangiopathies refer to chronic diseases of the bile ducts within the liver. Cholangiocytes—the epithelial cells lining the bile ducts—engage in the modification of bile volume and composition as well as in liver injury and repair [1]. All cholangiopathies are associated with bile flow obstruction, immune responses, and cholangiocyte proliferation, leading to biliary fibrosis, ductopenia, and eventually, biliary cirrhosis [2].

Cholangiopathies are classified into primary and secondary subtypes depending on whether the bile ducts are directly targeted (primary) or the bile duct degradation is a consequence of another pathological process or injury (secondary). Cholangiopathies lead to substantial morbidity and mortality due to the challenges linked with disease management and the lack of effective medical therapies [3]. The two major types of autoimmune cholestatic liver disease are primary biliary cholangitis (PBC) (previously known as primary biliary cirrhosis) and primary sclerosing cholangitis (PSC). These conditions are characterised by a sustained immune-mediated inflammatory response targeting cholangiocytes, which leads to the destruction of bile ducts and subsequent biliary stricturing due to excessive fibrotic deposition [4]. The progressive deterioration of the bile ducts causes impaired secretion and hepatic retention of bile toxins, which ultimately lead to biliary cirrhosis and hepatic failure requiring liver transplantation [5].

PBC is characterised by T-lymphocyte-mediated destruction of the intrahepatic small bile ducts [6], cholestatic liver biochemistries, and the presence of antimitochondrial antibodies (AMAs) [7]. These highly disease-specific autoantibodies are directed against the E2 subunit of the pyruvate dehydrogenase complex and are present in 90–95% of PBC patients and less than 1% of healthy controls [8,9]. In PSC, circulating autoantibodies are not as frequent, but rather auto-inflammation is a more typical characteristic of the condition [10]. Serological findings show non-specific immune abnormalities such as elevated levels of circulating immune complexes, immunoglobulins, non-organ specific autoantibodies, and T-lymphocyte infiltration within the portal tracts [11]. PSC radiological diagnostic images demonstrate a beaded pattern of both intra and extrahepatic bile ducts, and histological features suggest collagen fibre deposition around the bile ducts and infiltration of T cells [3,12].

The breakdown in peripheral tolerance to the biliary epithelium is a key driver of both PSC and PBC [13]. The strongest genetic associations of these diseases occupy distinct regions of the MHC (major histocompatibility complex), and most of the non-MHC associations are found in other autoimmune diseases, indicating altered immunoregulatory pathways [14]. In this review, we will discuss the role and interplay between regulatory T cells (Tregs) and mast cells (MCs) in the context of autoimmune cholangiopathies.

## 2. Treg Defects in Immune-Mediated Cholangiopathies

Tregs are crucial in the maintenance of peripheral tolerance and restraining aberrant immune responses. Self-reactive T cells reaching the periphery are subject to constant control and suppression by Tregs [15,16]. Tregs are defined by the expression of the core transcription factor FoxP3 [17,18,19], high levels of CD25 (the α-chain of the IL-2 receptor, and low levels of the IL-7 receptor (CD127) [20] (CD4^+^CD25^high^CD127^low^) (for reviews, see [21,22,23]).

The critical role of Tregs in the mediation of immune tolerance and prevention of autoimmunity is illustrated by the IPEX (immune dysregulation polyendocrinopathy enteropathy X-linked) syndrome, which is characterised by mutations in the human *FoxP3* gene that impair Treg development. In this condition, dysfunctional or deficient CD4^+^CD25^+^ Tregs cannot prevent the emergence of severe autoimmune and inflammatory diseases [24,25]. Similarly, Scurfy mice with a mutation in the *FoxP3* gene spontaneously develop fatal systemic autoimmune and inflammatory conditions [26].

Tregs are present within the human liver [27,28,29,30,31] and have been demonstrated to reside in portal tracts in proximity to effector CD4^+^ and CD8^+^ T cells. They play major roles in controlling liver inflammation, and the dysregulation of their quantities and functions has profound negative effects in the context of immune-mediated cholangiopathies [29]. The Treg to CD8^+^ effector T cell ratio was found to be lower in both blood and liver tissue of PBC patients compared to healthy controls [32,33], causing a subsequent breakdown in peripheral tolerance [32,33]. Evidence suggests that CD25 dysfunction may drive the persistence of autoreactive T cells in immune-mediated biliary disorders [34,35]. Human CD25 deficiency was observed to cause spontaneous development of a biliary condition [34], and CD25-deficient mice develop autoimmune cholangitis resembling human PBC [35]. Scurfy mice also exhibit PBC-like liver disease, characterised by bile duct damage induced by infiltrating autoreactive CD8^+^ T cells and upregulation of genes encoding pro-inflammatory cytokines [36].

In addition to Treg deficiencies, Th17 cells have been implicated in the emergence of inflammation and fibrosis in PBC [37,38,39,40]. Th17 cells are a unique CD4^+^ subset characterised by the production of the pro-inflammatory cytokine IL-17. This cytokine is a key driver of hepatic inflammation and fibrosis and is linked with the development of autoimmune liver diseases. IL-17 increases the production of inflammatory mediators (e.g., IL-6, IL-1β, TNFα) and pro-fibrotic mediators (e.g., Periostin, TGF-β, α-SMA), leading to collagen production [41].

The methylation status of the *FoxP3* locus determines the stability of the Treg population [42,43] and fine-tunes the Treg/Th17 balance [39]. The *FoxP3* promoter exhibits a highly methylated state in PBC patients compared to healthy patients and leads to a skewed Treg/Th17 differentiation axis towards Th17 cells [39]. The Th17 lineage-defining transcription factor RORγt is upregulated in PBC patients [39,40]. This upregulation causes dysregulation of the cytokine milieu in PBC with enrichment of pro-Th17 cytokines (IL-1β, IL-6, IL-23) and concurrent downregulation of FoxP3 and TGF-β expression in Tregs.

An analysis of the intrahepatic microenvironment demonstrated increased frequencies of TGF-β1 and IFN-γ in PBC livers, suggesting a role of these Th1 -related cytokines in PBC pathogenesis [44]. A negative correlation between CD4^+^CD25^+^ Tregs and IFN-γ was reported [44], signifying that the imbalance of CD4^+^CD25^+^ Tregs and cytotoxic cytokines may have important roles during PBC disease progression. Additionally, the inhibitory cytokine IL-35, which contributes to the suppressive properties of Tregs, is lower in PBC patients [45]. Plasma IL-35 concentration is lower in PBC patients than in healthy controls and negatively correlated to pro-inflammatory cytokine levels while being positively correlated to TGF-β concentration [46].

Tregs from PBC patients were also reported to have an increased sensitivity to IL-12 stimulation, even at low concentrations, which induces their differentiation into Th1-like cells (elevated levels of IFN-γ and T-bet expression) via STAT4 phosphorylation [47]. Tregs from PBC livers but not peripheral blood showed a significantly higher expression of IL-12Rβ2 compared to other cholestatic liver diseases and healthy controls [47]. This suggests that PBC Tregs have an increased plasticity tendency toward pro-inflammatory cell subsets, with the inflamed liver microenvironment driving this phenotype.

The transgenic dnTGFβRII mouse model, where both CD4^+^ and CD8^+^ T cells express a dominant negative type II TGF-β receptor transgene, also pointed out functional Treg defects. These mice spontaneously develop several features characteristic of human PBC [48]. In this model, Tregs exhibit defective suppressive functions rather than quantitative defects; they undergo phenotypic reprogramming towards a pro-inflammatory profile (Ccl5, Granzyme B and IFN-γ upregulation) and eventually promote autoimmunity [49,50]. Compensating this Treg defect via wild-type (WT) Treg adoptive transfer reduced disease severity in the dnTGFβRII models [51]. In contrast to the above-mentioned studies, the group of Gilberto Filaci revealed profound defects in the CD8-dependent regulatory pathway in PBC patients [52]. No reduction in the frequencies and numbers of circulating CD4^+^CD25^+^ and CD8^+^CD28^-^ Tregs were detected, and CD4^+^ Treg suppressive functions were not reduced. Nonetheless, the CD8^+^ Treg lineage exhibits not only a lesser in vitro generation capacity but also significantly lower immunosuppressive functional properties [52].

The role of Tregs in PSC is studied less extensively than in PBC [45,46]; however, a few studies have also highlighted Treg dysfunction. Christoph Schramm’s laboratory identified a significant decrease in circulating and intrahepatic Treg frequencies in PSC, as well as a reduced functionality (albeit the Treg dysfunction is limited) [53]. Without establishing a causal link, a low Treg number was associated with a polymorphism within the *IL2RA* gene locus [53]. Treatment of Mdr2^−/−^ mice with IL-2/IL-2ab complexes resulted in significantly higher numbers of Tregs in the liver [54]. This treatment caused a reduced production of IFN-γ and IL-17 and an increased IL-10 production by the isolated and re-stimulated liver lymphocytes. However, these changes had no significant effects on portal inflammation or fibrosis. The enriched hepatic Tregs displayed a significantly reduced suppressive function alongside lower FoxP3 expression and IL-12Rβ2 upregulation in comparison to splenic Tregs after in vivo expansion. This is likely due to the increased expression of IL-12 within the liver, which, in combination with the upregulation of IL-12Rβ2 in hepatic Tregs, may cause the reduced suppressive capacity in liver-derived Tregs [54]. In addition, IL-2 regulates liver Treg homeostasis in PSC by inducing CD39^+^ Tregs to suppress effector CD8^+^ T cell proliferation, thereby reducing biliary injury and fibrosis progression [55].

Over the last decade, advances in the knowledge of clinical-grade isolation reagents, cell-sorting strategies, and good manufacturing practice facilities have made GMP Treg infusion into patients a feasible therapy to restore peripheral tolerance in immune-mediated cholangiopathies [56]. So far, the above-mentioned in vitro and in vivo studies suggest that both PSC and PBC, would benefit from enhancing Treg frequency and function with GMP-Treg therapy alone or in combination with cytokine manipulation.

## 3. Hepatic Mast Cells and Their Roles in Immune-Mediated Cholangiopathies

MCs are multi-functional, tissue-resident immune cells that play multiple crucial roles in different inflammatory settings [57]. Previously, they were only known as effector cells; however, with ongoing research, it is now understood that they can exert immunomodulatory effects and enhance or suppress the initiation, scale, and/or duration of immune responses [58,59]. They originate from CD34^+^ hematopoietic stem cells and mature under the influence of cKIT ligand and stem cell factor (SCF) [59,60]; unlike most immune cells, MCs exit the bone marrow in an incompletely differentiated state and complete maturation within the tissue wherein they reside [61]. Different tissues of residence provide different maturation conditions, leading to phenotypical diversity in MCs. This heterogeneity is a result of the microenvironmental conditions that dictate gene expression and phenotypic development, resulting in biochemical, histochemical, and functional differences [62]. MCs are classified into two main subtypes: mucosal MCs that produce only tryptase and connective tissue MCs that produce both tryptase and chymase.

The cytoplasm of MCs has 50–200 large granules containing inflammatory mediators, which are released upon cell activation [57]—a process termed degranulation. Activation of MCs can occur via immune complexes (mostly via IgE engagement), complement products (C3a and C5a) [63], ligation of pattern receptors [64], certain neuropeptide receptors (such as acetylcholine, GABA, substance P, dopamine) [65], and venoms from poisonous animals [66]. During degranulation, MCs release an abundance of mediators, including growth factors (e.g., VEGF, FGF, TGF-β); cytokines (e.g., TNF, IFN-γ, IL-1, −2, −6, −10) and chemokines (e.g., CCL1, 2, 3, 4, 5, TGFβ, CXL2); as well as pro-inflammatory lipid mediators, such as prostaglandins and leukotrienes [67]. Pre-formed mediators from cytoplasmic granules include vasoactive amines (histamine and serotonin), proteoglycans (e.g., heparin), proteases (e.g., tryptases and chymases), as well as pre-stored cytokines (e.g., TNFα) [68]. Activation of MCs does not always cause degranulation, however, and MCs can release inflammatory mediators in situations where degranulation is not visible [69].

The knowledge of MC function within the human liver is still limited. Further fundamental studies characterising the biological activities of these complex cells are urgently needed to understand the role they play in not only liver homeostasis but also during inflammatory states such as in autoimmune disorders and infectious hepatitis. Several studies, however, have already highlighted an emerging role of MCs in autoimmune liver diseases.

**Figure 1:**
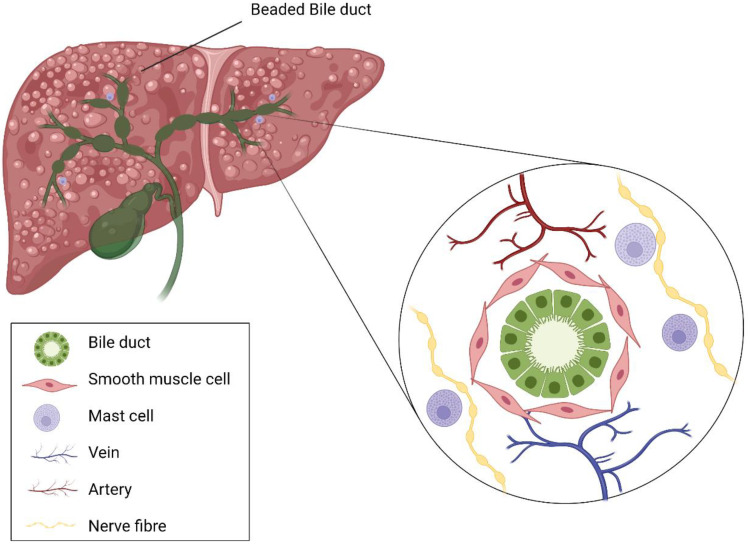
The location of mast cells within an inflamed liver. PSC/PBC livers share the pathological feature of extensive fibrosis. Beading of intra- and extra-hepatic bile ducts is a common feature in PSC and is due to collagen fibre deposition around the bile ducts, leading to compression. MCs have been observed to infiltrate areas of fibrosis and damaged bile ducts. Generally, MCs tend to associate with connective tissues found near hepatic arteries, veins, and bile ducts of the portal tracts. MCs reside close to vascular smooth muscle cells and may be involved in the regulation of the peribiliary vascular plexus along the intrahepatic biliary tree and in proximity to the nerve fibres around portal tracts. MC liver infiltration is significantly increased in immune-mediated cholangiopathies such as PSC and PBC.

MCs have been detected within the human liver [70] and tend to associate with connective tissues found near hepatic arteries, veins, and bile ducts of the portal tracts (known histologically as portal triads) [71,72,73]. In these areas, MCs were reported to reside in proximity to vascular smooth muscle cells [74] and may participate in the regulation of the peribiliary vascular plexus along the intrahepatic biliary tree, likely via chymase, endothelin-1, and nitric oxide signalling [74]. There is an anatomical association between MCs and neurons, and there have been multiple reports of MC and nerve crosstalk [75,76], with research suggesting that MCs act as important mediators between the immune and nervous systems. The liver is innervated by sympathetic and parasympathetic nerve fibres [77], and significantly higher numbers of MCs were observed near S100-positive nerve fibres in PBC in comparison to healthy livers [78] (Figure 1).

Numerous studies report increased numbers of MCs in cholangiopathies showing supportive evidence of MC involvement [71,79,80,81]. Both PSC and PBC share the pathological component of extensive fibrotic reactions, and MCs release a wide array of pro-fibrotic mediators (e.g., IL-1β, TNFα, TGFβ, FGF, histamine, tryptase, and chymase) [82]. All these factors are upregulated in cholestatic livers, and elevated histamine levels are associated with pruritus and trigger collagen production from fibroblasts [83,84,85]. MC numbers significantly increase in PBC livers in comparison to healthy and alcoholic livers, and their frequency correlates with the degree of liver fibrosis [72,79].Similar findings have been observed in PSC; cKIT-positive MCs infiltrate damaged bile ducts (identified to be SCF-positive), with a significantly higher infiltration of MCs reported in PSC than in chronic hepatitis C [80].

In vitro human studies are largely correlative, and therefore, in vivo murine models are utilised to demonstrate further evidence of MC influence in cholangiopathies. The two models commonly applied for functional studies are the Kit^W/Wv^ and Kit^w/sh^ mice models [86]. Both strains carry mutations in the cKIT receptor, resulting in major deficiencies in MC populations. The introduction of MCs into WT and Kit^w/sh^ mice induces chronic inflammation around the periportal area, leading to an increased ductular reaction, hepatic fibrosis, and biliary senescence [87]. TGF-β1 was identified as a crucial driver of this phenotype, and the injection of Kit^w/sh^ mice with MCs lacking TGF-β1 reversed these parameters. MC reintroduction did not seem to promote hepatocyte damage, thus demonstrating a preference for MC-cholangiocyte interactions. When bile duct ligation (BDL) was induced in Kit^w/sh^ mice, liver damage was reduced compared to WT mice with less proliferation, hepatic stellate cell (HSC) activation/fibrosis, and TGF-β1 expression/secretion [88]. These parameters were reversed in BDL Kit^w/sh^ mice after MC injection, providing supporting evidence of the role of MCs in cholangiopathies.

Histamine was highlighted as another important mediator in cholangiopathy progression. Mdr2^−/−^ mice treated with an MC stabiliser that blocks histamine release showed a significant reduction in biliary proliferation [70]. Immunohistochemistry and qPCR analyses revealed that treated mice had lower α-SMA, collagen type-1, and fibronectin levels, suggesting that MC-derived histamine may promote liver fibrosis. Decreased VEGF-α gene expression was also identified, demonstrating the role of histamine in vascular cell proliferation [70]. Co-culture experiments of HSCs and MCs conditioned to inhibit histamine production resulted in a significantly decreased proliferation of HSCs and a reduction in fibrosis markers and TGF-β1 [70]. These data suggest that MC-derived histamine alters HSC activation, driving fibrosis.

In addition, increased bile acid levels are a characteristic of PSC and PBC, and it has been shown that specific bile acids can cause MC activation in vitro and regulate histamine secretion. Ursodeoxycholic acid (UDCA)—a natural bile acid and a common treatment for biliary disorders—was found to reduce MC numbers, HSC activation, inflammation, and fibrosis in both Mdr2^−/−^ mice and PSC patients [89]. In vitro, UDCA decreased histamine release, and it was concluded that bile acids stimulate MC histamine release, which contributes to disease progression. Recently, it was shown that the Farnesoid X receptor (FXR)—also known as the bile acid receptor—regulates the ductular reaction in cholestasis [90]. Both biliary FXR and MC were upregulated during cholestatic liver disease, and the specific inhibition of MC-FXR signalling lessened the severity of cholestatic liver injury by reducing bile acid levels and reducing FXR/FGF15 signalling and histamine (HA/HRH1) signalling. These effects led to overall decreased biliary senescence.

## 4. Crosstalk between Mast Cells and Regulatory T Cells

Research on the crosstalk of Tregs and MCs within the hepatic environment is scarce; however, interactions have been identified in other tissues. Signalling can occur via several different mechanisms and appears to be bi-directional in nature. It is difficult to predict the pathways that occur between hepatic MCs and Tregs and how these pathways are dysregulated during cholangiopathies. Here, we will review some of the molecular interactions reported between MCs and Tregs and discuss the crosstalk and potential deregulation in the context of immune-mediated cholangiopathies.

Firstly, MCs are crucial mediators for Treg-mediated functions to occur. In certain models, MCs were shown to be protective in their interaction with Tregs. Inducible MHC class II expression by MCs gives them the ability to present antigens to Tregs directly and support their activation [91]. Tolerant allografts acquired a unique genetic signature with a high expression of MC gene signatures (MC protease 1 and 5, Tph1, FcεRI), with Tregs unable to maintain graft tolerance in MC-deficient mouse models [92]. The transfer of WT Tregs into Kit^w/wv^ mice completely prevented the shielding effects against the development of NTS (nephrotoxic serum nephritis), which normally occurs in WT mice [91,93].

One of the most detailed mechanisms of MC and Treg interactions is the OX40-OX40L pathway. Both Tregs and MCs constitutively express OX40 and OX40L, respectively [94,95]. The expression of both molecules increased in response to pro-inflammatory effects such as CD28 ligation and IFN-γ signalling [96]. Tregs directly suppress FcεRI-dependent MC degranulation through cell-to-cell contacts requiring OX40-OX40L interactions between MCs and Tregs [97]. This interaction leads to increased cAMP (cyclic adenosine monophosphate) levels and a reduced Ca^2+^ influx. Blocking cAMP in MCs reverses the inhibitory effects of Tregs and restores normal Ca^2+^ levels and degranulation. The depletion of Tregs in a mouse model of systemic anaphylaxis (an IgE-mediated hypersensitivity reaction involving MC degranulation) enhanced the extent of histamine release [97]. Considering that Treg depletion and functional defects are present in PSC and PBC, we can hypothesise that the increased histamine levels in these conditions may occur because of the lack of control of Tregs over MC degranulation.

Conversely, OX40/OX40L signalling between MCs and Tregs has also been found to suppress the regulatory effects of Tregs over T effector cells. Concurrent IL-6 abundance and scarcity of Th1/Th2 cytokines in the presence of activated MCs skews Tregs and effector T cells into Th17 cells through mechanisms involving OX40 engagement [98]. This feature is important to note since Th17 cells are associated with the development of autoimmune liver diseases [99,100]. The frequency of IL-17-secreting infiltrating cells was elevated in liver tissues of PBC patients and contributed to disease pathogenesis [101]. IL-6 can be produced by both MCs and Tregs; however, MCs only produce IL-6 in an IgE-sensitised and antigen-stimulated state, while effector T cells only produce IL-6 when stimulated by MCs, regardless of their activation or Treg presence. Interestingly, Treg suppression was only fully restored when both Tregs and effector T cells were OX40-deficient; this suggests that OX40 signalling may reduce effector T cell susceptibility to Treg suppression as well as directly hinder Treg functions. The abundance of IL-6 within an inflamed human liver [102] could be a contributing factor in enabling this pathway to occur. Tregs themselves can also enhance MC production of IL-6 in a contact-dependent manner via surface-bound TGFβ1 [103].

IL-2 is essential in the maintenance of Treg suppressive functions [104], and studies suggest MCs can regulate Treg expansion via IL-2 secretion. In a lung inflammation model, IL-33 induced IL-2 production by MCs, which resulted in Treg expansion and reduced inflammation [105]. In a skin inflammation model, IgE stimulation together with IL-33 enhanced IL-2 production by MCs [106]. A recent study identified that in an OIT (oral immunotherapy) murine model, desensitised MCs promote the expansion of Tregs via cytokine secretion [107]. Desensitisation of MCs is achieved by exposure to serially increasing doses of the relevant antigen, leading to temporary hyposensitivity to that antigen [108]. When CD4^+^ T cells were co-cultured with either desensitised or active, allergic MCs, the desensitised MCs could expand the Treg population. In this desensitised state, MCs secreted significantly higher amounts of IL-2 and IL-10, inducing FoxP3^+^ Tregs via an IL-33 independent mechanism [107]. The authors theorised that in the desensitised state, continuous stimulation of IgE by an antigen modulates FcεRI signal transduction, resulting in a functional change in MCs and the release of immunoregulatory mediators. The transfer of IL2^−/−^ MCs into Kit^w/sh^ mice did not dampen the inflammatory response in the same way as the transfer of WT MCs [109]. It was found that MC-derived IL-2 may be involved in maintaining the ratio between Tregs and effector T cells, and in the absence of IL-2, this ratio is skewed to promote effector T cell expansion. These studies highlight the importance of MC-derived IL-2 in the regulation of Treg functions in PBC and PSC.

Histamine was reported to directly affect Tregs and dampen their ability to suppress effector T cells. Higher histamine levels in co-culture experiments of histamine and Tregs led to a reduction in T cell effector suppression—an effect that was not observed in the absence of Tregs [110]. This occurred via H1 receptor stimulation on Tregs by histamine, which caused downregulation of CD25 and FoxP3 expression, thus limiting Treg-suppressive functions [110]. Constitutive CD25 expression on Tregs acts as a suppressive mechanism by outcompeting available IL-2 and inhibiting effector T cell proliferation [111]. Tregs can also suppress the release of histamine-preformed granules in vivo via the OX40/OX40L pathway in mouse models [97]. It is therefore plausible that the accumulation of MCs together with reduced Treg populations and functionality in PSC/PBC promotes histamine overproduction by MCs, which in turn further suppresses Treg function via the downregulation of FoxP3 and CD25.

Tregs have been shown to mediate the recruitment and functions of MCs via IL-9 production in vivo [92] and in NTS and gastric carcinoma models [112,113]. The neutralisation of IL-9 accelerated graft rejection in tolerant mice in skin transplantation models [92]. Co-localisation between SCF and IL-9 was identified in a tolerogenic liver allograft. This co-localisation was correlated with the activation of FoxP3 and IL-10 production, which resulted in the activation of hepatic MCs and histamine production [114]. These data suggest that hepatic Tregs may also be a source of SCF. A combination of IL-9 together with SCF could promote the proliferation of MCs from MC progenitors [115]. Moreover, IL-9 was shown to have crucial roles in the adoptive transfer of Tregs in NTS models with Tregs depending on IL-9 -mediated MC recruitment to conduct their immunosuppressive effects [112]. Therefore, IL-9 may be a crucial regulator of tolerance in immune-mediated cholangiopathies.

Treg-derived TGF-β1 has also been implied to play roles in the suppression of MC activation; in vitro assays revealed that TGF-β1 deficiency in Tregs impaired their capacity to suppress MC activation [116]. The same study found that the haploinsufficiency of TGF-β1 led to a significant gut tissue MC expansion and a steep increase in serum IgE concentrations. In line with the bi-directional nature of the interaction between MCs and Tregs, MC-derived TGF-β1 induces differentiation of CD4^+^ T cells into Tregs in vitro [117]. A positive feedback loop system has been reported through TGF-β1 and IL-9 in gastric carcinoma, with Tregs promoting MC proliferation via IL-9 and MCs inducing increased Tregs cells via TGF-β1 secretion. TGF-β1 production has also been observed in hepatic MCs [114], suggesting that a similar interaction may occur in the liver.

TNF-α stimulation enhances the immunoregulatory functions of murine MCs. More specifically, TNF-α treated MCs showed inhibited degranulation, which promoted CD4^+^ T cell differentiation to CD4^+^CD25^+^FoxP3^+^ T cells in co-culture studies, with MC degranulation inhibiting this effect [118]. The study also reported shifts in MC cytokine expression from a Th1 to a Th2 profile via a mechanism involving the upregulation of ICOSL (inducible co-stimulatory ligand) on MCs and MAPK phosphorylation. Studies have found that Th2 cytokines may play a role in the pathogenesis of PBC [119]. In PBC patients, elevated TNF-α levels were associated with greater disease severity, and UDCA treatments significantly reduce this cytokine [120]. Therefore, even though TNF-α stimulated MCs can increase Treg differentiation, the harmful Th2 effects may outweigh any protective functions.

Chymase inhibitors were found to be effective in reducing inflammation and led to a significant increase in the expression of immune-tolerance-related cytokines (IL-10, TGF-β1, IL-17A), FoxP3, and the amount of Tregs [121]. It is possible that MC-derived chymase is downregulating immune-tolerance related cytokines, thus inhibiting suppressive Treg functions; however, the mechanisms are not clear. Chymases can have a wide variety of substrates leading to a range of different effects. They may degrade harmful and pro-inflammatory substances or instead degrade protective molecules or even activate molecules that contribute to pathology [122]. The effects of chymases may depend on the availability of different chymase substrates, which are determined by the tissue and pathological setting. In the context of autoimmune diseases, mMCP-4 (mouse mast cell protease 4) contributes to the inflammatory response in arthritis and encephalomyelitis animal models [123,124]. Chymase was also reported to be increased in PBC livers [79]. MC-derived chymase may suppress Treg functions and/or recruitment, and thus, the increased chymase release due to MC accumulation in immune cholangiopathies worsens disease progression.

MCs exhibit cell plasticity, and cell cytokine signals, epigenetic changes, and other microenvironmental factors can rapidly and substantially alter their phenotype [125]. For example, IFN-γ stimulation of MCs caused a change in their signalling properties and in inflammatory conditions, with abundant IFN-γ MCs functioning as amplifiers of inflammation [121]. IFN-γ-primed MCs formed immunologic synapses with antigen-experienced CD4^+^ T cells. These interactions promoted the generation of Th22 and IL-22/IFN-γ producing cells via a TNF-α/IL-6 dependent mechanism [126]. This suggests that priming MCs towards a pro-inflammatory state may also promote Treg suppression.

**Figure 2:**
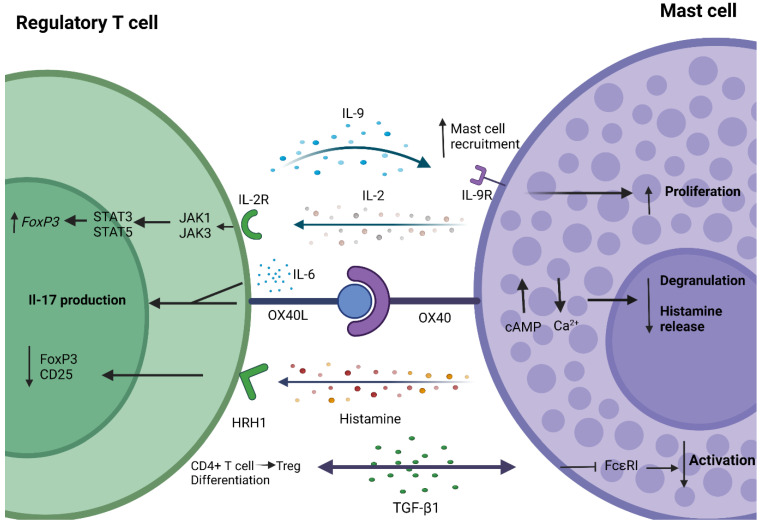
Molecular interactions between Regulatory T Cells (Tregs) and Mast Cells (MCs). Tregs can produce IL-9 to promote MC recruitment and proliferation via IL-9R engagment. MCs can secrete IL-2 and expand the Treg population via stimulation of IL-2R. This mechanism involves the activation of JAK1 and JAK3, then activating STAT3 and STAT5, which translocate into the nucleus to increase the expression of the *FoxP3* gene. The OX40/OX40L signalling pathway has bi-directional effects. OX40 stimulation on MCs increases expression of cAMP and decreases cellular Ca^2+^ influx; as a result, FcεRI-dependent MC degranulation is suppressed, which blocks histamine release. On Tregs, OX40L signalling together with IL-6 can skew Tregs into IL-17-producing Th17-like cells. Histamine secreted by MCs binds to Histamine H1 receptor (HRH1) on Tregs and directly downregulates FoxP3 and CD25. TGF-β1 secreted by Tregs can inhibit FcεRI signalling in MCs and prevent their activation. On the other hand, MC-derived TGF-β1 can promote the differentiation of CD4^+^ T cells into Tregs.

The described interactions of Treg and MCs (Figure 2) suggest bi-directional regulatory interactions between the cells. Tregs may regulate MC functions, and when Tregs become dysfunctional in cholangiopathies, this control may be lost. This, in turn, may shift the MC regulatory functions over Tregs from supportive to inhibitory and drive disease progression further. Modulating the MC phenotype may be crucial in restoring peripheral tolerance in autoimmune diseases.

## 5. Conclusions and Final Remarks

Controlling the untoward effector arm of the immune system is crucial in the treatment of cholangiopathies such as PBC and PSC. In the context of GMP Treg infusion therapy as an option for autoimmune liver diseases, it is critical to consider the effects of the inflammatory milieu of the liver and the potential reprogramming of the Treg phenotype towards effector lineages. The emerging role of MCs in the control of Tregs renders them an important innate cell population to consider in this context.

The reintroduction of the Treg population into the inflamed hepatic microenvironment may not be sufficient for restoring immune tolerance; the blockage of MC mediators, which suppress Treg functions or even promote their differentiation into pro-inflammatory, Th17-like cells, must also be considered. Modification of the hepatic microenvironment may be crucial to see the full benefits of Treg infusion therapy. Future research focusing on the interplay of these two cell types within the hepatic environment is needed to fully understand their emerging crosstalk in immune-mediated cholangiopathies.

## Data Availability

Not applicable.

## References

[1] Banales J.M., Huebert R.C., Karlsen T., Strazzabosco M., LaRusso N.F., Gores G.J. (2019). Cholangiocyte Pathobiology. Nat. Rev. Gastroenterol. Hepatol..

[2] Lazaridis K.N., LaRusso N.F. (2015). The Cholangiopathies. Mayo Clin. Proc..

[3] Tam P.K.H., Yiu R.S., Lendahl U., Andersson E.R. (2018). Cholangiopathies—Towards a Molecular Understanding. EBioMedicine.

[4] Giordano D., Pinto C., Maroni L., Benedetti A., Marzioni M. (2018). Inflammation and the Gut-Liver Axis in the Pathophysiology of Cholangiopathies. Int. J. Mol. Sci..

[5] Ronca V., Mancuso C., Milani C., Carbone M., Oo Y.H., Invernizzi P. (2020). Immune System and Cholangiocytes: A Puzzling Affair in Primary Biliary Cholangitis. J. Leukoc. Biol..

[6] Reshetnyak V.I. (2015). Primary Biliary Cirrhosis: Clinical and Laboratory Criteria for Its Diagnosis. World J. Gastroenterol..

[7] Yamagiwa S., Kamimura H., Takamura M., Aoyagi Y. (2014). Autoantibodies in Primary Biliary Cirrhosis: Recent Progress in Research on the Pathogenetic and Clinical Significance. World J. Gastroenterol..

[8] Janmohamed A., Trivedi P.J. (2018). Patterns of Disease Progression and Incidence of Complications in Primary Biliary Cholangitis (PBC). Best Pract. Res. Clin. Gastroenterol..

[9] Colapietro F., Lleo A., Generali E. (2021). Antimitochondrial Antibodies: From Bench to Bedside. Clin. Rev. Allergy Immunol..

[10] Strazzabosco M., Fiorotto R., Cadamuro M., Spirli C., Mariotti V., Kaffe E., Scirpo R., Fabris L. (2018). Pathophysiologic Implications of Innate Immunity and Autoinflammation in the Biliary Epithelium. Biochim. Biophys. Acta BBA-Mol. Basis Dis..

[11] Eaton J.E., Talwalkar J.A., Lazaridis K.N., Gores G.J., Lindor K.D. (2013). Pathogenesis of Primary Sclerosing Cholangitis and Advances in Diagnosis and Management. Gastroenterology.

[12] Khoshpouri P., Habibabadi R.R., Hazhirkarzar B., Ameli S., Ghadimi M., Ghasabeh M.A., Menias C.O., Kim A., Li Z., Kamel I.R. (2019). Imaging Features of Primary Sclerosing Cholangitis: From Diagnosis to Liver Transplant Follow-Up. RadioGraphics.

[13] Zhang H., Leung P.S.C., Gershwin M.E., Ma X. (2018). How the Biliary Tree Maintains Immune Tolerance?. Biochim. Biophys. Acta BBA-Mol. Basis Dis..

[14] Trivedi P.J., Hirschfield G.M. (2016). The Immunogenetics of Autoimmune Cholestasis. Clin. Liver Dis..

[15] Sakaguchi S., Sakaguchi N., Asano M., Itoh M., Toda M. (1995). Immunologic Self-Tolerance Maintained by Activated T Cells Expressing IL-2 Receptor Alpha-Chains (CD25). Breakdown of a Single Mechanism of Self-Tolerance Causes Various Autoimmune Diseases. J. Immunol..

[16] Sakaguchi S., Sakaguchi N., Shimizu J., Yamazaki S., Sakihama T., Itoh M., Kuniyasu Y., Nomura T., Toda M., Takahashi T. (2001). Immunologic Tolerance Maintained by CD25^+^ CD4^+^ Regulatory T Cells: Their Common Role in Controlling Autoimmunity, Tumor Immunity, and Transplantation Tolerance. Immunol. Rev..

[17] Fontenot J.D., Gavin M.A., Rudensky A.Y. (2003). Foxp3 Programs the Development and Function of CD4^+^CD25^+^ Regulatory T Cells. Nat. Immunol..

[18] Hori S., Nomura T., Sakaguchi S. (2003). Control of Regulatory T Cell Development by the Transcription Factor *Foxp3*. Science.

[19] Khattri R., Cox T., Yasayko S.-A., Ramsdell F. (2003). An Essential Role for Scurfin in CD4^+^CD25^+^ T Regulatory Cells. Nat. Immunol..

[20] Seddiki N., Santner-Nanan B., Martinson J., Zaunders J., Sasson S., Landay A., Solomon M., Selby W., Alexander S.I., Nanan R. (2006). Expression of Interleukin (IL)-2 and IL-7 Receptors Discriminates between Human Regulatory and Activated T Cells. J. Exp. Med..

[21] Terry L.V., Oo Y.H. (2020). The Next Frontier of Regulatory T Cells: Promising Immunotherapy for Autoimmune Diseases and Organ Transplantations. Front. Immunol..

[22] Sakaguchi S., Mikami N., Wing J.B., Tanaka A., Ichiyama K., Ohkura N. (2020). Regulatory T Cells and Human Disease. Annu. Rev. Immunol..

[23] Wing J.B., Tanaka A., Sakaguchi S. (2019). Human FOXP3^+^ Regulatory T Cell Heterogeneity and Function in Autoimmunity and Cancer. Immunity.

[24] Powell B.R., Buist N.R.M., Stenzel P. (1982). An X-Linked Syndrome of Diarrhea, Polyendocrinopathy, and Fatal Infection in Infancy. J. Pediatrics.

[25] Bacchetta R., Barzaghi F., Roncarolo M.-G. (2018). From IPEX Syndrome to *FOXP3* Mutation: A Lesson on Immune Dysregulation. Ann. N. Y. Acad. Sci..

[26] Godfrey V.L., Wilkinson J.E., Rinchik E.M., Russell L.B. (1991). Fatal Lymphoreticular Disease in the Scurfy (Sf) Mouse Requires T Cells That Mature in a Sf Thymic Environment: Potential Model for Thymic Education. Proc. Natl. Acad. Sci. USA.

[27] Longhi M.S., Mieli-Vergani G., Vergani D. (2021). Regulatory T Cells in Autoimmune Hepatitis: An Updated Overview. J. Autoimmun..

[28] Oo Y.H., Adams D.H. (2014). Regulatory T Cells and Autoimmune Hepatitis: What Happens in the Liver Stays in the Liver. J. Hepatol..

[29] Oo Y.H., Weston C.J., Lalor P.F., Curbishley S.M., Withers D.R., Reynolds G.M., Shetty S., Harki J., Shaw J.C., Eksteen B. (2010). Distinct Roles for CCR4 and CXCR3 in the Recruitment and Positioning of Regulatory T Cells in the Inflamed Human Liver. J. Immunol..

[30] Peiseler M., Sebode M., Franke B., Wortmann F., Schwinge D., Quaas A., Baron U., Olek S., Wiegard C., Lohse A.W. (2012). FOXP3+ Regulatory T Cells in Autoimmune Hepatitis Are Fully Functional and Not Reduced in Frequency. J. Hepatol..

[31] Taubert R., Hardtke-Wolenski M., Noyan F., Wilms A., Baumann A.K., Schlue J., Olek S., Falk C.S., Manns M.P., Jaeckel E. (2014). Intrahepatic Regulatory T Cells in Autoimmune Hepatitis Are Associated with Treatment Response and Depleted with Current Therapies. J. Hepatol..

[32] Lan R.Y., Cheng C., Lian Z.-X., Tsuneyama K., Yang G.-X., Moritoki Y., Chuang Y.-H., Nakamura T., Saito S., Shimoda S. (2006). Liver-Targeted and Peripheral Blood Alterations of Regulatory T Cells in Primary Biliary Cirrhosis. Hepatology.

[33] Chen J., Hou X., Jia H., Cui G., Wu Z., Wang L., Lu C., Wu W., Wei Y., Uede T. (2017). Regulatory T Cells with a Defect in Inhibition on Co-Stimulation Deteriorated Primary Biliary Cholangitis. Oncotarget.

[34] Aoki C.A., Roifman C.M., Lian Z.-X., Bowlus C.L., Norman G.L., Shoenfeld Y., Mackay I.R., Eric Gershwin M. (2006). IL-2 Receptor Alpha Deficiency and Features of Primary Biliary Cirrhosis. J. Autoimmun..

[35] Wakabayashi K., Lian Z.-X., Moritoki Y., Lan R.Y., Tsuneyama K., Chuang Y.-H., Yang G.-X., Ridgway W., Ueno Y., Ansari A.A. (2006). IL-2 Receptor A−/− Mice and the Development of Primary Biliary Cirrhosis. Hepatology.

[36] Zhang W., Sharma R., Ju S.-T., He X.-S., Tao Y., Tsuneyama K., Tian Z., Lian Z.-X., Fu S.M., Gershwin M.E. (2009). Deficiency in Regulatory T Cells Results in Development of Antimitochondrial Antibodies and Autoimmune Cholangitis. Hepatology.

[37] Yang C.-Y., Ma X., Tsuneyama K., Huang S., Takahashi T., Chalasani N.P., Bowlus C.L., Yang G.-X., Leung P.S.C., Ansari A.A. (2014). IL-12/Th1 and IL-23/Th17 Biliary Microenvironment in Primary Biliary Cirrhosis: Implications for Therapy. Hepatology.

[38] Cichoż-Lach H., Grywalska E., Michalak A., Kowalik A., Mielnik M., Roliński J. (2018). Deviations in Peripheral Blood Cell Populations Are Associated with the Stage of Primary Biliary Cholangitis and Presence of Itching. Arch. Immunol. Ther. Exp..

[39] Jiang T., Zhang H., Wen Y., Yin Y., Yang L., Yang J., Lan T., Tang C., Yu J., Tai W. (2021). 5-Aza-2-Deoxycytidine Alleviates the Progression of Primary Biliary Cholangitis by Suppressing the FoxP3 Methylation and Promoting the Treg/Th17 Balance. Int. Immunopharmacol..

[40] Rong G., Zhou Y., Xiong Y., Zhou L., Geng H., Jiang T., Zhu Y., Lu H., Zhang S., Wang P. (2009). Imbalance between T Helper Type 17 and T Regulatory Cells in Patients with Primary Biliary Cirrhosis: The Serum Cytokine Profile and Peripheral Cell Population. Clin. Exp. Immunol..

[41] Beringer A., Miossec P. (2018). IL-17 and IL-17-Producing Cells and Liver Diseases, with Focus on Autoimmune Liver Diseases. Autoimmun. Rev..

[42] Floess S., Freyer J., Siewert C., Baron U., Olek S., Polansky J., Schlawe K., Chang H.-D., Bopp T., Schmitt E. (2007). Epigenetic Control of the Foxp3 Locus in Regulatory T Cells. PLoS Biol..

[43] Helmin K.A., Morales-Nebreda L., Torres Acosta M.A., Anekalla K.R., Chen S.-Y., Abdala-Valencia H., Politanska Y., Cheresh P., Akbarpour M., Steinert E.M. (2020). Maintenance DNA Methylation Is Essential for Regulatory T Cell Development and Stability of Suppressive Function. J. Clin. Investig..

[44] Wang D., Zhang H., Liang J., Gu Z., Zhou Q., Fan X., Hou Y., Sun L. (2010). CD4^+^CD25^+^ but Not CD4^+^Foxp3^+^ T Cells as a Regulatory Subset in Primary Biliary Cirrhosis. Cell. Mol. Immunol..

[45] Collison L.W., Workman C.J., Kuo T.T., Boyd K., Wang Y., Vignali K.M., Cross R., Sehy D., Blumberg R.S., Vignali D.A.A. (2007). The Inhibitory Cytokine IL-35 Contributes to Regulatory T-Cell Function. Nature.

[46] Li T., Huang Y., Liu P., Liu Y., Guo J., Zhang W., Gu M., Qian C., Deng A. (2018). Lower Plasma Levels of IL-35 in Patients with Primary Biliary Cirrhosis. Tohoku J. Exp. Med..

[47] Liaskou E., Patel S.R., Webb G., Bagkou Dimakou D., Akiror S., Krishna M., Mells G., Jones D.E., Bowman S.J., Barone F. (2018). Increased Sensitivity of Treg Cells from Patients with PBC to Low Dose IL-12 Drives Their Differentiation into IFN-γ Secreting Cells. J. Autoimmun..

[48] Oertelt S., Lian Z.-X., Cheng C.-M., Chuang Y.-H., Padgett K.A., He X.-S., Ridgway W.M., Ansari A.A., Coppel R.L., Li M.O. (2006). Anti-Mitochondrial Antibodies and Primary Biliary Cirrhosis in TGF-β Receptor II Dominant-Negative Mice. J. Immunol..

[49] Huang W., Kachapati K., Adams D., Wu Y., Leung P.S.C., Yang G.-X., Zhang W., Ansari A.A., Flavell R.A., Gershwin M.E. (2014). Murine Autoimmune Cholangitis Requires Two Hits: Cytotoxic KLRG1^+^ CD8 Effector Cells and Defective T Regulatory Cells. J. Autoimmun..

[50] Wang Y.-H., Yang W., Yang J.-B., Jia Y.-J., Tang W., Gershwin M.E., Ridgway W.M., Lian Z.-X. (2015). Systems Biologic Analysis of T Regulatory Cells Genetic Pathways in Murine Primary Biliary Cirrhosis. J. Autoimmun..

[51] Tanaka H., Zhang W., Yang G.-X., Ando Y., Tomiyama T., Tsuneyama K., Leung P., Coppel R.L., Ansari A.A., Lian Z.X. (2014). Successful Immunotherapy of Autoimmune Cholangitis by Adoptive Transfer of Forkhead Box Protein 3^+^ Regulatory T Cells. Clin. Exp. Immunol..

[52] Bernuzzi F., Fenoglio D., Battaglia F., Fravega M., Gershwin M.E., Indiveri F., Ansari A.A., Podda M., Invernizzi P., Filaci G. (2010). Phenotypical and Functional Alterations of CD8 Regulatory T Cells in Primary Biliary Cirrhosis. J. Autoimmun..

[53] Sebode M., Peiseler M., Franke B., Schwinge D., Schoknecht T., Wortmann F., Quaas A., Petersen B.-S., Ellinghaus E., Baron U. (2014). Reduced FOXP3+ Regulatory T Cells in Patients with Primary Sclerosing Cholangitis Are Associated with IL2RA Gene Polymorphisms. J. Hepatol..

[54] Schwinge D., von Haxthausen F., Quaas A., Carambia A., Otto B., Glaser F., Höh B., Thiele N., Schoknecht T., Huber S. (2017). Dysfunction of Hepatic Regulatory T Cells in Experimental Sclerosing Cholangitis Is Related to IL-12 Signaling. J. Hepatol..

[55] Taylor A.E., Carey A.N., Kudira R., Lages C.S., Shi T., Lam S., Karns R., Simmons J., Shanmukhappa K., Almanan M. (2018). Interleukin 2 Promotes Hepatic Regulatory T Cell Responses and Protects From Biliary Fibrosis in Murine Sclerosing Cholangitis. Hepatology.

[56] Jeffery H.C., Braitch M.K., Brown S., Oo Y.H. (2016). Clinical Potential of Regulatory T Cell Therapy in Liver Diseases: An Overview and Current Perspectives. Front. Immunol..

[57] Krystel-Whittemore M., Dileepan K.N., Wood J.G. (2016). Mast Cell: A Multi-Functional Master Cell. Front. Immunol..

[58] Reber L.L., Sibilano R., Mukai K., Galli S.J. (2015). Potential Effector and Immunoregulatory Functions of Mast Cells in Mucosal Immunity. Mucosal Immunol..

[59] Gri G., Frossi B., D’Inca F., Danelli L., Betto E., Mion F., Sibilano R., Pucillo C. (2012). Mast Cell: An Emerging Partner in Immune Interaction. Front. Immunol..

[60] Shimizu Y., Sakai K., Miura T., Narita T., Tsukagoshi H., Satoh Y., Ishikawa S., Morishita Y., Takai S., Miyazaki M. (2002). Characterization of ‘Adult-Type’ Mast Cells Derived from Human Bone Marrow CD34+ Cells Cultured in the Presence of Stem Cell Factor and Interleukin-6. Interleukin-4 Is Not Required for Constitutive Expression of CD54, FcεRIα and Chymase, and CD13 Expressi. Clin. Exp. Allergy.

[61] Gurish M.F., Austen K.F. (2012). Developmental Origin and Functional Specialization of Mast Cell Subsets. Immunity.

[62] Moon T.C., St Laurent C.D., Morris K.E., Marcet C., Yoshimura T., Sekar Y., Befus A.D. (2010). Advances in Mast Cell Biology: New Understanding of Heterogeneity and Function. Mucosal Immunol..

[63] Elieh Ali Komi D., Shafaghat F., Kovanen P.T., Meri S. (2020). Mast Cells and Complement System: Ancient Interactions between Components of Innate Immunity. Allergy.

[64] Agier J., Pastwińska J., Brzezińska-Błaszczyk E. (2018). An Overview of Mast Cell Pattern Recognition Receptors. Inflamm. Res..

[65] Xu H., Shi X., Li X., Zou J., Zhou C., Liu W., Shao H., Chen H., Shi L. (2020). Neurotransmitter and Neuropeptide Regulation of Mast Cell Function: A Systematic Review. J. Neuroinflamm..

[66] Galli S.J., Starkl P., Marichal T., Tsai M. (2016). Mast Cells and IgE in Defense against Venoms: Possible “Good Side” of Allergy?. Allergol. Int..

[67] Paivandy A., Pejler G. (2021). Novel Strategies to Target Mast Cells in Disease. J. Innate Immun..

[68] Bulfone-Paus S., Bahri R. (2015). Mast Cells as Regulators of T Cell Responses. Front. Immunol..

[69] Galli S.J., Nakae S., Tsai M. (2005). Mast Cells in the Development of Adaptive Immune Responses. Nat. Immunol..

[70] Jones H., Hargrove L., Kennedy L., Meng F., Graf-Eaton A., Owens J., Alpini G., Johnson C., Bernuzzi F., Demieville J. (2016). Inhibition of Mast Cell-Secreted Histamine Decreases Biliary Proliferation and Fibrosis in Primary Sclerosing Cholangitis Mdr2^−/−^ Mice. Hepatology.

[71] Nakamura A., Yamazaki K., Suzuki K., Sato S. (1997). Increased Portal Tract Infiltration of Mast Cells and Eosinophils in Primary Biliary Cirrhosis. Am. J. Gastroenterol..

[72] Farrell D.J., Hines J.E., Walls A.F., Kelly P.J., Bennett M.K., Burt A.D. (1995). Intrahepatic Mast Cells in Chronic Liver Diseases. Hepatology.

[73] Jarido V., Kennedy L., Hargrove L., Demieville J., Thomson J., Stephenson K., Francis H. (2017). The Emerging Role of Mast Cells in Liver Disease. Am. J. Physiol. -Gastrointest. Liver Physiol..

[74] Koda W., Harada K., Tsuneyama K., Kono N., Sasaki M., Matsui O., Nakanuma Y. (2000). Evidence of the Participation of Peribiliary Mast Cells in Regulation of the Peribiliary Vascular Plexus Along the Intrahepatic Biliary Tree. Lab. Investig..

[75] Mittal A., Sagi V., Gupta M., Gupta K. (2019). Mast Cell Neural Interactions in Health and Disease. Front. Cell. Neurosci..

[76] Siiskonen H., Harvima I. (2019). Mast Cells and Sensory Nerves Contribute to Neurogenic Inflammation and Pruritus in Chronic Skin Inflammation. Front. Cell. Neurosci..

[77] Mizuno K., Ueno Y. (2017). Autonomic Nervous System and the Liver. Hepatol. Res..

[78] Matsunaga Y., Kawasaki H., Terada T. (1999). Stromal Mast Cells and Nerve Fibers in Various Chronic Liver Diseases: Relevance to Hepatic Fibrosis. Am. J. Gastroenterol..

[79] Satomura K., Yin M., Shimizu S., Kato Y., Nagano T., Komeichi H., Ohsuga M., Katsuta Y., Aramaki T., Omoto Y. (2003). Increased Chymase in Livers with Autoimmune Disease: Colocalization with Fibrosis. J. Nippon Med. Sch..

[80] Ishii M., Iwai M., Harada Y., Morikawa T., Okanoue T., Kishikawa T., Tsuchihashi Y., Hanai K., Arizono N. (2005). A Role of Mast Cells for Hepatic Fibrosis in Primary Sclerosing Cholangitis. Hepatol. Res..

[81] Tsuneyama K., Kono N., Yamashiro M., Kouda W., Sabit A., Sasaki M., Nakanuma Y. (1999). Aberrant Expression of Stem Cell Factor on Biliary Epithelial Cells and Peribiliary Infiltration of C-Kit-Expressing Mast Cells in Hepatolithiasis and Primary Sclerosing Cholangitis: A Possible Contribution to Bile Duct Fibrosis. J. Pathol..

[82] Weiskirchen R., Meurer S.K., Liedtke C., Huber M. (2019). Mast Cells in Liver Fibrogenesis. Cells.

[83] Gittlen S.D., Schulman E.S., Maddrey W.C. (1990). Raised Histamine Concentrations in Chronic Cholestatic Liver Disease. Gut.

[84] Abe M., Yokoyama Y., Amano H., Matsushima Y., Kan C., Ishikawa O. (2002). Effect of Activated Human Mast Cells and Mast Cell-Derived Mediators on Proliferation, Type I Collagen Production and Glycosaminoglycans Synthesis by Human Dermal Fibroblasts. Eur. J. Dermatol..

[85] González M.I., Vannan D.T., Eksteen B., Flores-Sotelo I., Reyes J.L. (2022). Mast Cells in Immune-Mediated Cholangitis and Cholangiocarcinoma. Cells.

[86] Reber L.L., Marichal T., Galli S.J. (2012). New Models for Analyzing Mast Cell Functions in Vivo. Trends Immunol..

[87] Kyritsi K., Kennedy L., Meadows V., Hargrove L., Demieville J., Pham L., Sybenga A., Kundu D., Cerritos K., Meng F. (2021). Mast Cells Induce Ductular Reaction Mimicking Liver Injury in Mice Through Mast Cell–Derived Transforming Growth Factor Beta 1 Signaling. Hepatology.

[88] Hargrove L., Kennedy L., Demieville J., Jones H., Meng F., DeMorrow S., Karstens W., Madeka T., Greene J., Francis H. (2017). Bile Duct Ligation-Induced Biliary Hyperplasia, Hepatic Injury, and Fibrosis Are Reduced in Mast Cell-Deficient Kit W-Sh Mice. Hepatology.

[89] Meng F., Kennedy L., Hargrove L., Demieville J., Jones H., Madeka T., Karstens A., Chappell K., Alpini G., Sybenga A. (2018). Ursodeoxycholate Inhibits Mast Cell Activation and Reverses Biliary Injury and Fibrosis in Mdr2^−/−^ Mice and Human Primary Sclerosing Cholangitis. Lab. Investig..

[90] Meadows V., Kennedy L., Ekser B., Kyritsi K., Kundu D., Zhou T., Chen L., Pham L., Wu N., Demieville J. (2021). Mast Cells Regulate Ductular Reaction and Intestinal Inflammation in Cholestasis Through Farnesoid X Receptor Signaling. Hepatology.

[91] Kambayashi T., Allenspach E.J., Chang J.T., Zou T., Shoag J.E., Reiner S.L., Caton A.J., Koretzky G.A. (2009). Inducible MHC Class II Expression by Mast Cells Supports Effector and Regulatory T Cell Activation. J. Immunol..

[92] Lu L.-F., Lind E.F., Gondek D.C., Bennett K.A., Gleeson M.W., Pino-Lagos K., Scott Z.A., Coyle A.J., Reed J.L., van Snick J. (2006). Mast Cells Are Essential Intermediaries in Regulatory T-Cell Tolerance. Nature.

[93] Eller K., Wolf D., Huber J.M., Metz M., Mayer G., McKenzie A.N.J., Maurer M., Rosenkranz A.R., Wolf A.M. (2011). IL-9 Production by Regulatory T Cells Recruits Mast Cells That Are Essential for Regulatory T Cell-Induced Immune Suppression. J. Immunol..

[94] Sibilano R., Frossi B., Suzuki R., D’Incà F., Gri G., Piconese S., Colombo M.P., Rivera J., Pucillo C.E. (2012). Modulation of FcεRI-Dependent Mast Cell Response by OX40L via Fyn, PI3K, and RhoA. J. Allergy Clin. Immunol..

[95] Valzasina B., Guiducci C., Dislich H., Killeen N., Weinberg A.D., Colombo M.P. (2005). Triggering of OX40 (CD134) on CD4+CD25+ T Cells Blocks Their Inhibitory Activity: A Novel Regulatory Role for OX40 and Its Comparison with GITR. Blood.

[96] Webb G.J., Hirschfield G.M., Lane P.J.L. (2016). OX40, OX40L and Autoimmunity: A Comprehensive Review. Clin. Rev. Allergy Immunol..

[97] Gri G., Piconese S., Frossi B., Manfroi V., Merluzzi S., Tripodo C., Viola A., Odom S., Rivera J., Colombo M.P. (2008). CD4^+^CD25^+^ Regulatory T Cells Suppress Mast Cell Degranulation and Allergic Responses through OX40-OX40L Interaction. Immunity.

[98] Piconese S., Gri G., Tripodo C., Musio S., Gorzanelli A., Frossi B., Pedotti R., Pucillo C.E., Colombo M.P. (2009). Mast Cells Counteract Regulatory T-Cell Suppression through Interleukin-6 and OX40/OX40L Axis toward Th17-Cell Differentiation. Blood.

[99] Behfarjam F., Nasseri-Moghaddam S., Jadali Z. (2019). Enhanced Th17 Responses in Patients with Autoimmune Hepatitis. Middle East J. Dig. Dis..

[100] Drescher H.K., Bartsch L.M., Weiskirchen S., Weiskirchen R. (2020). Intrahepatic TH17/TReg Cells in Homeostasis and Disease—It’s All About the Balance. Front. Pharmacol..

[101] Harada K., Shimoda S., Sato Y., Isse K., Ikeda H., Nakanuma Y. (2009). Periductal Interleukin-17 Production in Association with Biliary Innate Immunity Contributes to the Pathogenesis of Cholangiopathy in Primary Biliary Cirrhosis. Clin. Exp. Immunol..

[102] Johnson C., Han Y., Hughart N., McCarra J., Alpini G., Meng F. (2012). Interleukin-6 and Its Receptor, Key Players in Hepatobiliary Inflammation and Cancer. Transl. Gastrointest. Cancer.

[103] Ganeshan K., Bryce P.J. (2012). Regulatory T Cells Enhance Mast Cell Production of IL-6 via Surface-Bound TGF-β. J. Immunol..

[104] Chinen T., Kannan A.K., Levine A.G., Fan X., Klein U., Zheng Y., Gasteiger G., Feng Y., Fontenot J.D., Rudensky A.Y. (2016). An Essential Role for the IL-2 Receptor in Treg Cell Function. Nat. Immunol..

[105] Morita H., Arae K., Unno H., Miyauchi K., Toyama S., Nambu A., Oboki K., Ohno T., Motomura K., Matsuda A. (2015). An Interleukin-33-Mast Cell-Interleukin-2 Axis Suppresses Papain-Induced Allergic Inflammation by Promoting Regulatory T Cell Numbers. Immunity.

[106] Salamon P., Shefler I., Moshkovits I., Munitz A., Horwitz Klotzman D., Mekori Y.A., Hershko A.Y. (2017). IL-33 and IgE Stimulate Mast Cell Production of IL-2 and Regulatory T Cell Expansion in Allergic Dermatitis. Clin. Exp. Allergy.

[107] Takasato Y., Kurashima Y., Kiuchi M., Hirahara K., Murasaki S., Arai F., Izawa K., Kaitani A., Shimada K., Saito Y. (2021). Orally Desensitized Mast Cells Form a Regulatory Network with Treg Cells for the Control of Food Allergy. Mucosal Immunol..

[108] Ang W.X.G., Church A.M., Kulis M., Choi H.W., Burks A.W., Abraham S.N. (2016). Mast Cell Desensitization Inhibits Calcium Flux and Aberrantly Remodels Actin. J. Clin. Investig..

[109] Hershko A.Y., Suzuki R., Charles N., Alvarez-Errico D., Sargent J.L., Laurence A., Rivera J. (2011). Mast Cell Interleukin-2 Production Contributes to Suppression of Chronic Allergic Dermatitis. Immunity.

[110] Forward N.A., Furlong S.J., Yang Y., Lin T.-J., Hoskin D.W. (2009). Mast Cells Down-Regulate CD4+CD25+ T Regulatory Cell Suppressor Function via Histamine H1 Receptor Interaction. J. Immunol..

[111] de la Rosa M., Rutz S., Dorninger H., Scheffold A. (2004). Interleukin-2 Is Essential for CD4+CD25+ Regulatory T Cell Function. Eur. J. Immunol..

[112] Wolf A.M., Wolf D., McKenzie A., Maurer M., Rosenkranz A.R., Eller K. (2010). IL-9 Production by Regulatory T Cells Recruits Mast Cells That Are Essential for Regulatory T Cell-Induced Immune-Suppression. Blood.

[113] Zhao Y.-B., Yang S.-H., Shen J., Deng K., Li Q., Wang Y., Cui W., Ye H. (2020). Interaction between Regulatory T Cells and Mast Cells via IL-9 and TGF-&Beta; Production. Oncol. Lett..

[114] Nakano T., Lai C.-Y., Goto S., Hsu L.-W., Kawamoto S., Ono K., Chen K.-D., Lin C.-C., Chiu K.-W., Wang C.-C. (2012). Immunological and Regenerative Aspects of Hepatic Mast Cells in Liver Allograft Rejection and Tolerance. PLoS ONE.

[115] Matsuzawa S., Sakashita K., Kinoshita T., Ito S., Yamashita T., Koike K. (2003). IL-9 Enhances the Growth of Human Mast Cell Progenitors Under Stimulation with Stem Cell Factor. J. Immunol..

[116] Turner J.A., Stephen-Victor E., Wang S., Rivas M.N., Abdel-Gadir A., Harb H., Cui Y., Fanny M., Charbonnier L.-M., Fong J.J.H. (2020). Regulatory T Cell-Derived TGF-Β1 Controls Multiple Checkpoints Governing Allergy and Autoimmunity. Immunity.

[117] Zhang W., Wu K., He W., Gao Y., Huang W., Lin X., Cai L., Fang Z., Zhou Q., Luo Z. (2010). Transforming Growth Factor Beta 1 Plays an Important Role in Inducing CD4+CD25+forhead Box P3+ Regulatory T Cells by Mast Cells. Clin. Exp. Immunol..

[118] Gao Y., Xu B., Zhang P., He Y., Liang X., Liu J., Li J. (2017). TNF-α Regulates Mast Cell Functions by Inhibiting Cell Degranulation. Cell. Physiol. Biochem..

[119] Nagano T., Yamamoto K., Matsumoto S., Okamoto R., Tagashira M., Ibuki N., Matsumura S., Yabushita K., Okano N., Tsuji T. (1999). Cytokine Profile in the Liver of Primary Biliary Cirrhosis. J. Clin. Immunol..

[120] Neuman M., Angula P., Malkiewicz I., Jorgensen R., Shear N., Dickson E.R., Haber J., Katz G., Lindor K. (2002). Tumor Necrosis Factor-α and Transforming Growth Factor-β Reflect Severity of Liver Damage in Primary Biliary Cirrhosis. J. Gastroenterol. Hepatol..

[121] Liu W.-X. (2016). Chymase Inhibitor TY-51469 in Therapy of Inflammatory Bowel Disease. World J. Gastroenterol..

[122] Pejler G. (2020). Novel Insight into the in Vivo Function of Mast Cell Chymase: Lessons from Knockouts and Inhibitors. J. Innate Immun..

[123] Magnusson S.E., Pejler G., Kleinau S., Åbrink M. (2009). Mast Cell Chymase Contributes to the Antibody Response and the Severity of Autoimmune Arthritis. FASEB J..

[124] Desbiens L., Lapointe C., Gharagozloo M., Mahmoud S., Pejler G., Gris D., D’Orléans-Juste P. (2016). Significant Contribution of Mouse Mast Cell Protease 4 in Early Phases of Experimental Autoimmune Encephalomyelitis. Mediat. Inflamm..

[125] Galli S.J., Borregaard N., Wynn T.A. (2011). Phenotypic and Functional Plasticity of Cells of Innate Immunity: Macrophages, Mast Cells and Neutrophils. Nat. Immunol..

[126] Gaudenzio N., Laurent C., Valitutti S., Espinosa E. (2013). Human Mast Cells Drive Memory CD4^+^ T Cells toward an Inflammatory IL-22+ Phenotype. J. Allergy Clin. Immunol..

